# The evolution of computed tomography from organ-selective to whole-body scanning in managing unconscious patients with multiple trauma: A retrospective cohort study

**DOI:** 10.1097/MD.0000000000004653

**Published:** 2016-09-16

**Authors:** Zhi-Jie Hong, Cheng-Jueng Chen, Jyh-Cherng Yu, De-Chuan Chan, Yu-Ching Chou, Chia-Ming Liang, Sheng-Der Hsu

**Affiliations:** a General Surgery, Department of Surgery, Tri-Service General Hospital, National Defense Medical Center; b Trauma Surgery, Department of Surgery, Tri-Service General Hospital, National Defense Medical Center; c Graduate Institute of Medical Sciences, National Defense Medical Center; d School of Public Health, National Defense Medical Center, Taipei, Taiwan, ROC.

**Keywords:** Multiple trauma, selective-organ CT, whole-body CT

## Abstract

We aimed to evaluate the benefit of whole-body computed tomography (WBCT) scanning for unconscious adult patients suffering from high-energy multiple trauma compared with the conventional stepwise approach of organ-selective CT.

Totally, 144 unconscious patients with high-energy multiple trauma from single level I trauma center in North Taiwan were enrolled from January 2009 to December 2013. All patients were managed by a well-trained trauma team and were suitable for CT examination. The enrolled patients are all transferred directly from the scene of an accident, not from other medical institutions with a definitive diagnosis. The scanning regions of WBCT include head, neck, chest, abdomen, and pelvis. We analyzed differences between non-WBCT and WBCT groups, including gender, age, hospital stay, Injury Severity Score, Glasgow Coma Scale, Revised Trauma Score, time in emergency department (ED), medical cost, and survival outcome.

Fifty-five patients received the conventional approach for treating trauma, and 89 patients received immediate WBCT scanning after an initial examination. Patients’ time in ED was significantly shorter in the WBCT group in comparison with the non-WBCT group (158.62 ± 80.13 vs 216.56 ± 168.32 min, *P* = 0.02). After adjusting for all possible confounding factors, we also found that survival outcome of the WBCT group was better than that of the non-WBCT group (odds ratio: 0.21, 95% confidence interval: 0.06–0.75, *P* = 0.016).

Early performing WBCT during initial trauma management is a better approach for treating unconscious patients with high-energy multiple trauma.

## 1 Introduction

Over half of patients die within 24 h after major multiple trauma including blunt abdominal or thoracic trauma with massive bleeding, or severe primary brain injury.^[1]^ The detectable rate of an initial physical examination for intra-abdominal injury decreases to 16% in patients with unconsciousness after multiple trauma involving head injuries.^[2–5]^ Moreover, it is difficult to completely exclude abdominal or pelvic organ injury based on physical examination or conventional radiographic images, including plain films of the chest, pelvis, lateral projection of the cervical spine, or focused assessment sonography in trauma (FAST) according to Advanced Trauma Life Support (ATLS) standards.^[6]^ The efficacy and benefit of organ-selective computed tomography (CT) for managing patients with blunt thoracic or abdominal trauma with stable hemodynamic conditions has been well documented after a fast, initial examination.^[7–11]^

We require 1 suspected severely traumatized region after a primary survey to order organ-selective CT for managing a patient with multiple trauma. This requirement seems more suitable for managing patients with a solitary region of trauma than those with severe multiple trauma. In patients with unconsciousness, organ-selective CT seems insufficient to evaluate potentially lethal injuries. Therefore, the current trend is to apply whole-body computed tomography (WBCT) increasingly on managing patients with multiple severe injuries and unconsciousness. It seems to improve the likelihood of survival.^[12–17]^

However, WBCT has also been considered having more unnecessary radiation exposure compared with initial plain films or organ-selective CT, thus possibly increasing cancer incidence. But we still have no evidence to negate the theory. So we have to evaluate the clinical outcomes of patients who underwent or did not undergo WBCT (WBCT and non-WBCT groups, respectively) to determine whether the benefits of WBCT scanning to patients with multiple trauma and decreased consciousness can overcome the risk of increased radiation exposure. This study aimed to determine the effect of immediate and fast WBCT scanning compared with standard conventional radiological imaging on the clinical outcomes of patients with multiple trauma.

## 2 Methods

**Figure 1:**
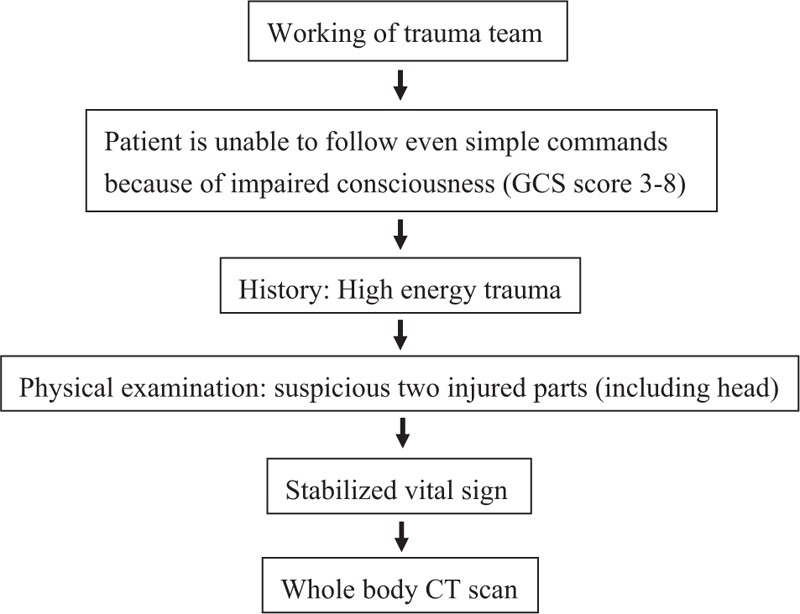
Protocol of performing whole-body computed tomography scan.

The Institutional Review Board of Tri-Service General Hospital, National Defense Medical Center approved this retrospective study without written informed consent. All patient records and information in the registered trauma database had been anonymized and deidentified prior to analysis. The approval number is TSGHIRB No. 2-103-05-106. Our hospital is a level I trauma center in Taipei, Taiwan, staffed with in-house attending physicians and equipped with appropriate facilities to manage patients with severe multisystem trauma. A trauma registry established by our hospital in 2011 is used to analyze treatment strategies and factors that influence the care of severely injured patients, and it serves as a tool for the management and monitoring of the quality of care. At the time the present study commenced, the database contained records of 5242 patients with trauma. Data show that the fast 256-slice WBCT has been routinely performed in unconscious patients with multiple trauma and stable vital signs since January 2012 according to the protocol of performing WBCT scan that was established in the same time (Fig. 1). The regions of WBCT scanning include the head, neck, chest, abdomen, and pelvis. Before introducing this management approach, we only performed organ-selective CT in such patients after conducting a primary survey and a conventional imaging study.

We enrolled patients age at least 20 years who had multiple trauma and received conventional organ-selective CT (non-WBCT group, n = 55) or WBCT using the 256-slice system (WBCT group, n = 89) who were treated from January 2011 to December 2013. These patients were managed by a trauma team comprising surgeons with primary expertise in the following surgical disciplines, chief resident of general, thoracic, cardiovascular, genitourinary, neurological, and orthopedic as well as the physician attending trauma team leader on duty. The trauma team treated patients meeting the following criteria: unconsciousness (patient is unable to follow even simple commands) and Glasgow Coma Scale (GCS) < 8 points; initial hemodynamic instability, systolic blood pressure < 90 mm Hg; fell from a height of 6 m or from a second floor; gunshot to the head and trunk; severe pelvic fracture; and suspected multiple injuries to vital organs.

The exclusion criteria were any one of the following: patients transferred from other medical institutions with a definitive diagnosis, penetrating injury without blunt trauma, died in emergency department (ED), incomplete conventional radiologic work-up (including organ-selective CT or WBCT scanning) due to persistent hemodynamic instability after adequate resuscitation.

This collected data about unconscious patients suffered from multiple trauma are based on the working of trauma team. Loss of some cases may happen without the working of trauma team. This may be the selected bias from our study design.

Results are expressed as mean ± standard deviation. Analysis of variance was used to analyze the confounding effects of age, hospital stay, Revised Trauma Score (RTS), Injury Severity Score (ISS), total hospital cost, and time in ED on the clinical outcomes of the non-WBCT group compared with those of the WBCT group. Gender and outcome (mortality or not) were compared using the chi-squared test. A multivariate logistic regression model was used to adjust for all confounding factors to decrease interactional bias. Statistical analysis was performed using SPSS Version 17.0 software for Windows (SPSS Inc., Chicago, IL). A *P* value ≤ 0.05 was considered statistically significant.

## 3 Results

The 55 patients in the non-WBCT group underwent standard conventional radiological imaging (including conventional organ-selected CT) from January 2011 to December 2011 according to the ATLS guidelines. The 89 patients in the WBCT group underwent fast WBCT scanning immediately after a primary survey from January 2012 to December 2013 according to the protocol (Fig. 1). The patients’ characteristics are presented in Table 1. The non-WBCT group spent a significantly longer time in ED compared with the WBCT group. There were no significant differences between the 2 groups in age, gender, hospital stay, ISS, GCS, RTS, and survival outcome (Table 1). Further, there was no significant difference in costs for treating the 2 groups (*P* = 0.388), but there was a decreasing trend of the costs for treating the WBCT group compared with the non-WBCT group (Table 1).

**Table 1:**
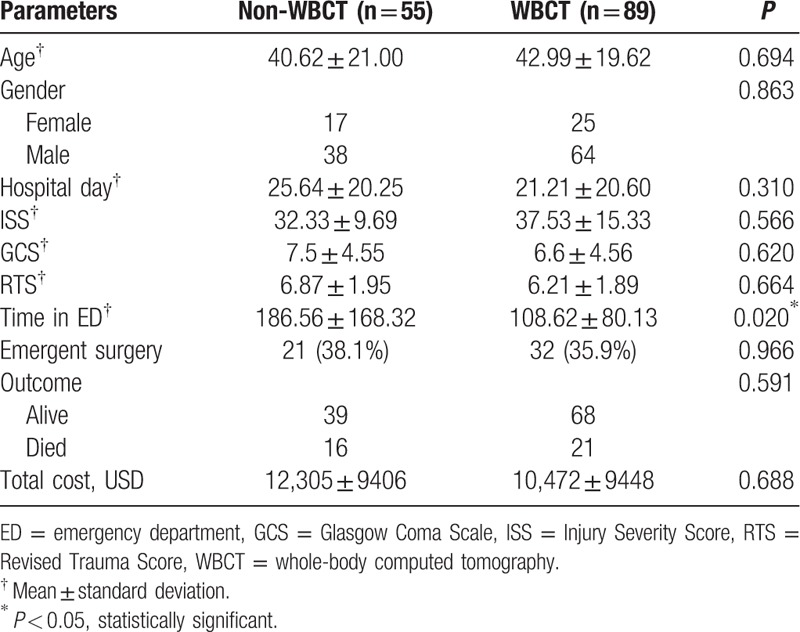
Characteristics of patients in the non-WBCT and WBCT groups (n = 144).

Because these confounding factors had more of an effect on group interaction, we used multivariate logistic regression model to adjust for all parameters. We found that hospital stay, time in ED, and mortality rates among patients in the WBCT group were all significantly lower compared with those of patients in the non-WBCT group (odds ratio [OR]: 0.9788, 95% confidence interval [CI]: 0.9581–0.9999, *P* = 0.049; OR: 0.9956, 95% CI: 0.9917–0.9995, *P* = 0.029; OR: 0.21, 95% CI: 0.06–0.75, *P* = 0.016, respectively) (Table 2).

**Table 2:**
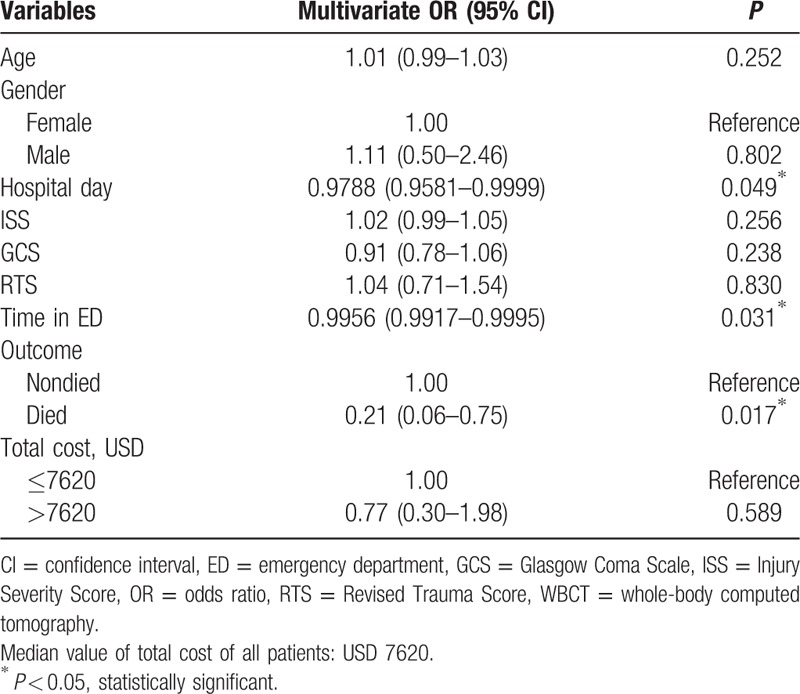
Multivariate logistic regression analysis of confounding factors between WBCT and non-WBCT groups.

After admission, we performed different follow-up examinations according to each patient's new complaints, symptoms, and signs. There were more undetected injuries to sites in the non-WBCT group compared with those in the WBCT group as follows: intracranial hemorrhage (5.4% vs 1.1%), C-spinal/L-spinal injury (3.6% vs 0%), rib fracture (7.2% vs 0%), hemo/pneumothorax (3.6% vs 1.1%), abdominal injury (14.4% vs 1.1%), pelvic bone fracture (3.6% vs 0%), major vessel injury (1.8% vs 0%), and musculoskeletal injury (5.4% vs 0%) (Table 3). Patients in the non-WBCT group required treatment more frequently compared with those in the WBCT group for injuries to the organs as follows: bowel (3.6% vs 1.1%), internal iliac artery (1.8% vs 0%), and femoral neck fracture (3.6% vs 0%) (Table 4).

**Table 3:**
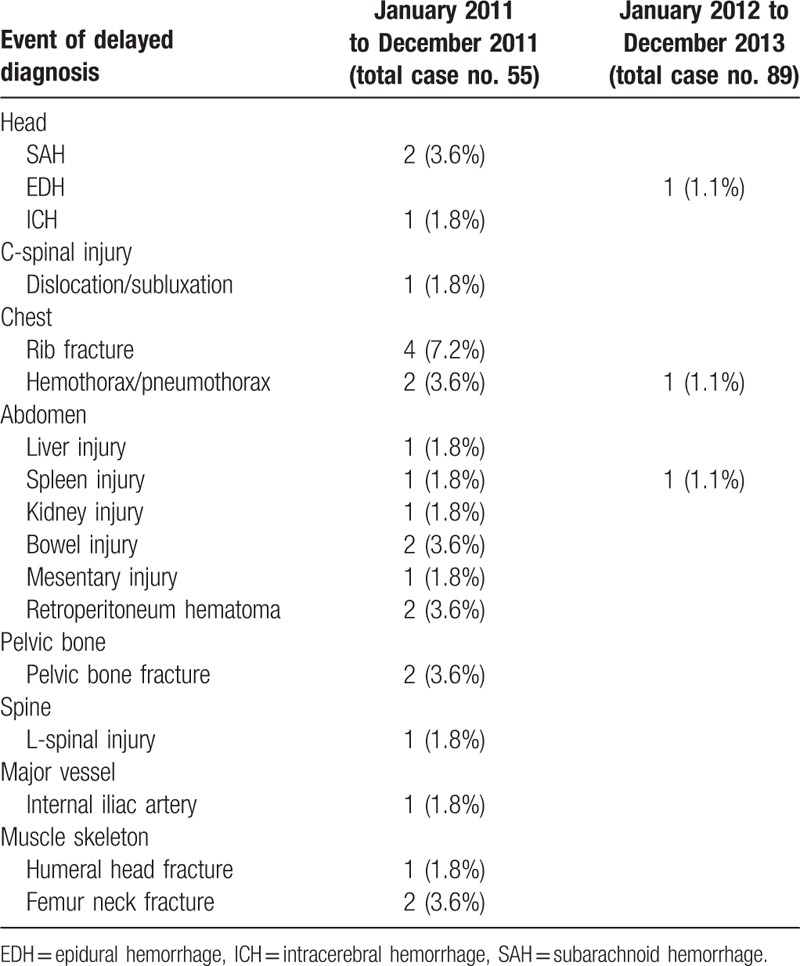
Results of follow-up studies of patients after admission.

**Table 4:**
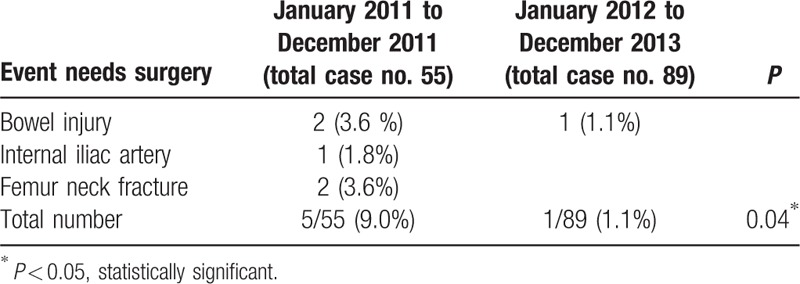
Cases of delayed diagnosis need surgery.

## 4 Discussion

According to ATLS,^[6]^ CT of the spine may be used as the method of choice for radiographic assessment of an unconscious patient who requires CT of the brain after suffering multiple trauma. Many trauma centers now use CT instead plain films for detecting spinal injury during secondary surveys. Some literatures had suggested that the application of CT can be as a screening and diagnostic tool, so CT could be used widely to replace traditional radiography on managing trauma.^[18,19]^ Managing unconscious patients with undetermined thoracic and abdominal examination results is one of the indications for WBCT.^[20]^

We started to perform WBCT frequently on unconscious patients with suspected injuries to multiple organs when the concept of WBCT was introduced in our hospital since January 2012. Initially, the cost effect of WBCT on patient with multiple trauma was in doubt because of some studies report that WBCT or CT of the torso may increase radiation exposure without decreased mortality.^[21,22]^ In the review of literatures, typical doses from a single WBCT examination are approximately 16 mGy to the lung, 14 mGy to the digestive organs, and 10 mGy to the bone marrow. The effective dose, which is the weighted average of doses to all organs, is approximately 12 mSv.^[23]^ In general, WBCT is associated with greater radiation exposure compared with organ-selective CT, and it potentially increases an individual's risk of cancer. To estimate radiation exposure, an effective radiation dose is assumed to range from 10 to 20 mSv for a WBCT, 5 to 16 mSv for a selective-organ CT, and 2 mSv for a conventional radiography series (chest, vertebral column, and pelvis). The radiation exposure in this subpopulation undergoing our WBCT scan protocol is about 50 mSv according to CT technician's estimation. As to the attributable risk of cancer in the lifetime, there is no definite estimation in this subpopulation until now. In our review, for a multiphase abdomen and pelvis CT, the median effective dose was 31 mSv, and for a 20-year-old woman, the corresponding median adjusted lifetime attributable risk of cancer was 4 cancers per 1000 patients (range = 0.83–11.1 cancers per 1000 patients).^[24]^ So we only can estimate the attributable risk of cancer is more than 0.4 % in our subpopulation. Until now, only 1 study reported the cancer mortality risk of WBCT in detail. The lifetime and attributable cancer mortality risk of a single WBCT examination of a 45-year-old patient is about 0.08%.^[25]^ In our study, although we did not estimate the potentially harmful effects of increased radiation exposure in detail, the mortality risk ratio in the WBCT group compared with that in the non-WBCT group was reduced by a factor of approximately 0.79 (reduced OR) (Table 2).

Fast, accurate, and comprehensive diagnosis is still the first mandatory criterion in unconscious, critically injured patients, although having the small increased risk of developing cancer years or decades later. Therefore, we consider WBCT as an important management tool in unconscious patients with multiple trauma after compared the benefit of reduced immediate mortality with the risk of cancer mortality in the future.

For blunt abdominal trauma, CT is more sensitive and specific than diagnostic peritoneal lavage or FAST for detecting free intraperitoneal fluid or air, and it may detect active contrast extravasation from vascular or visceral injuries. Moreover, if we identified clinically stable patients with proven hepatic and splenic injuries by intravenous contrast-enhanced CT, these patients can be treated conservatively by trans-arterial embolization or monitoring in intensive care unit.^[26]^ Further, trauma team leader can save the time on negotiating with radiologists to gain access to CT data after the conventional approach step by step. Fewer return visits for imaging can greatly reduce the associated life-threatening risks because of the transfer and delays in the definitive management of injured patients.

In our study, based on the same level of ISS and RTS, the patients have significantly reduced mortality risk when they received WBCT compared with non-WBCT. The time spent in ED in the WBCT group was also significantly shorter compared with that in the non-WBCT group statistically (Table 1). Therefore, we concluded shorter time spent in ED due to shortening the time to arrive at a decision for managing patients can attribute to increasing patients’ survival rate and a delay in performing appropriate surgery is a major cause of preventable deaths in trauma management. The earliest possible identification of potential lethal organ injuries is mandatory for optimal trauma care.^[27–30]^ Therefore, early detection and definite management for multiple trauma ensure more successful outcomes.

In the review of literatures, there is a significant reduction in the time from the patient's arrival at ED to the initiation of emergency surgery when compared a multislice CT (MSCT)-based trauma algorithm with a conventional approach.^[31]^ Emergency surgery initiated after a mean time of 103 min in their MSCT cohort. In our present study, the WBCT group spent a mean time of 108 min in ED. A significant reduction in treatment time in ED was also reported when using an MSCT-based trauma algorithm.^[32]^ There is a 40% reduction in diagnostic work-up time using MSCT without first performing plain film radiography.^[33]^ Our studies, taken together with other studies cited here, provide strong evidence that WBCT will become the first modality of choice worldwide for managing unconscious patients with multiple trauma.

Another benefit of WBCT for multiple trauma is significantly decreasing the case number of delayed diagnoses of injured sites. In our present study, the number of delayed diagnosis is greatly decreased from 25 (non-WBCT) to 3 (WBCT). Approximately 22 events in the non-WBCT group may have been detected earlier if WBCT had been performed, including head, neck, chest, abdominal, and pelvic injuries after excluding 3 instances of musculoskeletal injuries (1 humeral head and 2 femoral neck fractures). However, there were 3 delayed diagnoses in the WBCT group, which we attributed to delayed epidural hemorrhage (EDH), delayed pneumothorax, and delayed bowel perforation. The number of delayed diagnosis of life-threatening events is also decreased from 5 (non-WBCT) to 1 (WBCT) and reached statistical significance (*P* = 0.04 < 0.05). Besides, there is significantly increased number of unnecessary scan in the WBCT group (Table 5), especially in the abdominal and pelvic scan (*P* = 0.047 < 0.05). But, because of the high frequency of medical malpractice disputes, WBCT offer to promise to greatly decrease the risk of medical staff from suffering legal problems in our country.

**Table 5:**
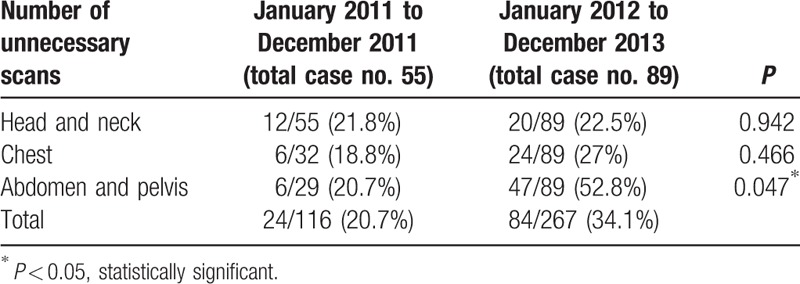
Number of unnecessary scans: compare with these 2 groups.

In clinical practice, more examinations involving imaging studies would lead to higher costs for maintaining an ED. In our study, we found that the cost incurred by the WBCT group was not significantly higher compared with that incurred by the non-WBCT group. In contrast, the cost of WBCT tended to decrease compared with that of non-WBCT (Table 1).

The main strength in this study is to supply a strong evidence that the WBCT scan for a unconscious patient with multiple trauma is suitable and safe if we can follow a good and standard protocol of performing CT in trauma cases. The limitations are that randomized control study is difficult to be carried out in an emergent moment in our country because a detail work-up and medical information supplied are always requested by any patient's family arrived at ED.

## 5 Conclusions

The WBCT scanning could be recommended as a standard diagnostic approach for initial trauma care after adequate fluid resuscitation in these comatose patients. It promises to significantly increase the survival probability of comatose patients with multiple trauma.
